# Recurrent neural network based high-precision position compensation control of magnetic levitation system

**DOI:** 10.1038/s41598-022-15638-0

**Published:** 2022-07-06

**Authors:** Zhiwen Huang, Jianmin Zhu, Jiajie Shao, Zhouxiang Wei, Jiawei Tang

**Affiliations:** 1 School of Mechanical Engineering, University of Shanghai for Science and Technology, Shanghai, 200093 China; 2 School of Mechanical Engineering, Tongji University, Shanghai, 200092 China

**Keywords:** Computer science, Electrical and electronic engineering

## Abstract

For improving the dynamic quality and steady-state performance, the hybrid controller based on recurrent neural network (RNN) is designed to implement the position control of the magnetic levitation ball system in this study. This hybrid controller consists of a baseline controller, an RNN identifier, and an RNN controller. In the hybrid controller, the baseline controller based on the control law of proportional-integral-derivative is firstly employed to provide the online learning sample and maintain the system stability at the early control phase. Then, the RNN identifier is trained online to learn the accurate inverse model of the controlled object. Next, the RNN controller shared the same structures and parameters with the RNN identifier is applied to add the precise compensation control quantity in real-time. Finally, the effectiveness and advancement of the proposed hybrid control strategy are comprehensively validated by the simulation and experimental tests of tracking step, square, sinusoidal, and trapezoidal signals. The results indicate that the RNN-based hybrid controller can obtain higher precision and faster adjustment than the comparison controllers and has strong anti-interference ability and robustness.

## Introduction

Benefiting from its superiority of the contactless, frictionless, and noiseless, magnetic levitation system has been successfully applied to many fields^1,2^, such as high-speed maglev trains^3^, maglev wind turbines^4^, and frictionless magnetic bearings^5^. With the open-loop unstable and inherent nonlinear characteristics, it is a constant challenge to design the high-performance control scheme for position control of the magnetic levitation system^6,7^.

Over the years, various control strategies have been successively developed to implement real-time position control of the magnetic levitation system. The control strategies mainly include feedback linearization control^8,9^, proportional-integral-derivative (PID) control^10,11^, model predictive control^12,13^, robust H-infinity control^14,15^, sliding mode control^16,17^, and adaptive fuzzy control^18,19^. Although these different control strategies can achieve good control results from different perspectives^7^, there is still room for improvement in the control performance of the magnetic levitation system to a certain extent.

Recently, the intelligent control based on artificial neural networks (ANNs) can obtain a better tracking performance and disturbance rejection, benefiting from ANNs’ excellent ability to learn the dynamic model of the nonlinear control system^20^. Correspondingly, ANNs can improve the position control performance of the magnetic levitation system due to its powerful self-learning and adaptive abilities. For example, Rubio et al. presented a neural network controller consisting of a nonlinear method and a neural network and reduced the root mean square error of the trajectory tracking in the magnetic levitation system^21^. Silva et al. constructed a neural controller to magnetic levitation system and obtained a better control performance than the classical controllers^22^. Wei et al. proposed a feedback compensation controller based on backpropagation neural network (BPNN), which lowered the steady-state error in position control of the magnetic levitation ball system^23^. Yang et al. designed an adaptive sliding mode controller (SMC) based on radial basis function neural network (RBFNN) to the magnetic levitation system and acquired faster convergence and stronger robustness than the traditional SMC method^24^. Sahoo et al. applied the functional link artificial neural network to the Fuzzy PID controller and validated its superiority in the real-time Maglev control system^25^. Tang et al. presented a hybrid controller based on BPNN and fuzzy inference for the magnetic levitation ball system, which improved the dynamical response of tracking step and square signals^26^. Qin et al. designed a model predictive controller based on RBFNN to control the position of the maglev ball, which obtained a better track performance of the step signal than conventional PID controller^27^. Sun et al. presented a supervisor control method based on the RBF neural network and achieved an excellent tracking performance in the flexible and time-delay control of the maglev train system^28^.

Through the above literature review, it can be found that these neural networks used in the control systems belong to the feedforward neural networks, which can improve the control performance of the magnetic levitation system by approximating a nonlinear function without prior knowledge of the closed-loop control system^26^. However, the parameter updating of these feedforward neural networks reckon without the historical information of the control system, which limits the nonlinear approximation of the controlled object to some extent, thereby hindering the further improvement in the control performance of the magnetic levitation system.

The recurrent neural network (RNN) can acquire the dynamic response of the control system by using delays of an internal feedback loop, which has the ability to deal with the time-varying control system, especially in nonlinear and uncertain scenarios^29,30^. Lin et al. designed a hybrid computed force control method with an RNN uncertainty observer, which improved the tracking accuracy in position control of the magnetic levitation system^30^. Fatemimoghadam et al. proposed an adaptive backstepping control scheme based on a projection RNN for the magnetic levitation system, which achieved a better control performance than the sliding mode control method^31^. Jafari and Hagan applied RNN to the model reference control of the magnetic levitation system and obtained better performance than linear PID controllers^32^. Hou et al. embedded a recurrent feature selection neural network to an intelligent global sliding mode controller (GSMC) and acquired superior performance than traditional GSMC^33^.

Therefore, motived by the mentioned superiorities of the RNN in the controller design of the nonlinear time-varying system, a hybrid controller based on RNN is proposed to improve the control performance in the position control of the magnetic levitation ball system in this article. The main contributions are summarized in the following.

1. An intelligent controller based on the accurate compensation control is developed to ensure both dynamical performance and steady-state performance of the control system, which consists of a PID-based baseline controller, an RNN identifier, and an RNN controller.
2. The RNN identifier is designed to online learn the inverse model of the control system, and the learning parameters are passed to the RNN controller sharing the same structure as the RNN identifier in real-time, which achieves the accurate compensation control.
3. The simulation analysis and experimental verification are shown comprehensively to prove the effectiveness and advancement of the proposed RNN-based intelligent controller and the improvement of the transient performance and the steady-state accuracy with a certain robustness.

The remainder of this article is organized as follows. Section “Problem formulation” formalizes the problem to be solved. Then, the hybrid controller is specifically designed in Section “Controller design”. Next, simulation analysis and experimental verification are presented in Sections “Simulation analysis” and “Experimental verification” respectively. Finally, Sections “Discussion” and “Conclusion” give the conclusion of this study and discuss the potential future work.

## Problem formulation

**Figure 1:**
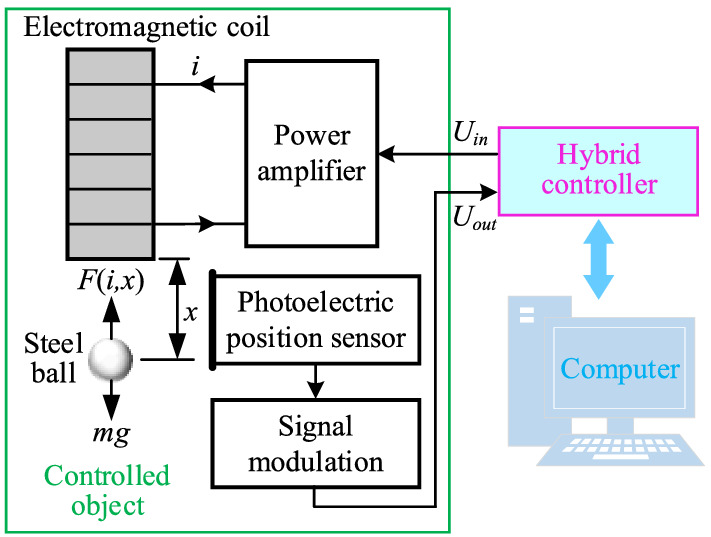
Schematic diagram of the control system physical entity.

This study focuses on the position control of a magnetic levitation ball system, and the schematic diagram of its physical entity is shown in Fig. 1. The system is mainly composed of an electromagnetic coil, a steel ball, a power amplifier, a photoelectric position sensor, a data acquisition and control card, and a computer. In this system, the input voltage of the power amplifier is controlled to make the coil current generate the appropriate electromagnetic force, thereby the position of the steel ball can be controlled.

To describe the position control problem of the magnetic levitation ball system, as shown in Fig. 1, let $F(i,x)$ be the electromagnetic force at the current $i$ of the electromagnetic coil and the position $x$ of the steel ball, $U_{in}$ represents the input voltage of the power amplifier, $m$ and $g$ denote the mass of the steel ball and the gravitational acceleration respectively. In addition, this study supposes that there is no magnetic flux leakage and iron coil magnetoresistance, the electromagnetic force of the steel ball is concentrated at the center of its gravity, and the output current of the power amplifier has a linear relationship with its input voltage. Concretely, the mathematical model of the control object can be established in the following.

First, the steel ball is only subject to the electromagnetic force $F(i,x)$ and its gravity $mg$ in the controlled system, ignoring other interference forces on the ball. Thus the dynamic equation of the steel ball in the vertical direction can be described as follows

1 $$m\ddot{x} = F(i,x) + mg$$

where $\ddot{x} \triangleq d^{2} x(t)/dt^{2}$ denotes the acceleration of the ball at position $x$.

Then, based on Kirchoff’s law and Biot-Savart’s law, the electromagnetic force $F(i,x)$ can be calculated as follows^22^

2 $$F(i,x) = K \cdot (i/x)^{2}$$

where $K = \mu_{0} AN^{2} /4$, and parameters $\mu_{0}$, $A$, and $N$ represent the vacuum permeability, permeability area, and coil turn respectively.

When the ball is in the equilibrium position, its acceleration is zero and can be expressed as

3 $$f(i_{0} ,x_{0} ) + mg = 0$$

where $F(i_{0} ,x_{0} )$ denotes the electromagnetic force at the equilibrium point $(i_{0} ,x_{0} )$.

Next, according to Faraday's law and Kirchoff’s law, the relationship between control voltage and current in the electromagnet winding can be expressed as follows

4 $$U_{in} = Ri + L \cdot di/dt$$

where $R$ and $L$ are the resistance and self-inductance of the electromagnetic coil respectively.

Finally, the magnetic levitation ball system can be described jointly by the above equations, and the expression is as follows

5 $$\left\{ {\begin{array}{*{20}l} {m\ddot{x} = F(i,x) + mg} \hfill \\ {F(i,x) = K \cdot (i/x)^{2} } \hfill \\ {F(i_{0} ,x_{0} ) + mg = 0} \hfill \\ {U_{in} = Ri + L \cdot di/dt} \hfill \\ \end{array} } \right.$$

Expanding the Taylor series at the equilibrium point $(i_{0} ,x_{0} )$, the electromagnetic force $F(i,x)$ is equivalent to

6 $$F(i,x) = F(i_{0} ,x_{0} ) + K_{i} (i - i_{0} ) + K_{x} (x - x_{0} ) + F_{h} (i,x)$$

where $K_{i} \triangleq F_{i} (i_{0} ,x_{0} ) = \tfrac{\partial F}{{\partial i}}|_{{(i_{0} ,x_{0} )}} = {{2K \cdot i_{0} } \mathord{\left/ {\vphantom {{2K \cdot i_{0} } {x_{0}^{2} }}} \right. \kern-\nulldelimiterspace} {x_{0}^{2} }}$, $K_{x} \triangleq F_{x} (i_{0} ,x_{0} ) = \tfrac{\partial F}{{\partial x}}|_{{(i_{0} ,x_{0} )}} = {{ - 2K \cdot i_{0}^{2} } \mathord{\left/ {\vphantom {{ - 2K \cdot i_{0}^{2} } {x_{0}^{3} }}} \right. \kern-\nulldelimiterspace} {x_{0}^{3} }}$, and $F_{h} (i,x)$ is the remaining high-order term of $F(i,x)$.

When omitting the high-order term, the kinetic equation of the steel ball can be rewritten as

7 $$\begin{aligned} m\ddot{x} & = F(i_{0} ,x_{0} ) + K_{i} (i - i_{0} ) + K_{x} (x - x_{0} ) + mg \\ & = K_{i} (i - i_{0} ) + K_{x} (x - x_{0} ){\kern 1pt} {\kern 1pt} = ({{2Ki_{0} } \mathord{\left/ {\vphantom {{2Ki_{0} } {x_{0}^{2} }}} \right. \kern-\nulldelimiterspace} {x_{0}^{2} }})i - ({{2Ki_{0}^{2} } \mathord{\left/ {\vphantom {{2Ki_{0}^{2} } {x_{0}^{3} }}} \right. \kern-\nulldelimiterspace} {x_{0}^{3} }})x \\ \end{aligned}$$

For the magnetic levitation ball system, the input voltage $U_{in}$ of the power amplifier is the input of the control system, while the output voltage $U_{out}$ of the sensor module is the output of the control system. Taking the Laplace transformation of Eq. (7), the open-loop control system can be calculated by

8 $$G(s) = \frac{{U_{out} (s)}}{{U_{in} (s)}} = \frac{{K_{s} x(s)}}{{K_{a} i(s)}} = \frac{{K_{s} (2Ki_{0} /mx_{0}^{2} )}}{{K_{a} (s^{2} + (2Ki_{0}^{2} /mx_{0}^{3} ))}}$$

Substitute the boundary equation $mg = - F(i_{0} ,x_{0} ) = - K \cdot ({{i_{0} } \mathord{\left/ {\vphantom {{i_{0} } {x_{0} }}} \right. \kern-\nulldelimiterspace} {x_{0} }})^{2}$ to Eq. (8), then the open-loop control system can be written as

9 $$G(s) = \frac{{ - ({{K_{s} } \mathord{\left/ {\vphantom {{K_{s} } {K_{a} }}} \right. \kern-\nulldelimiterspace} {K_{a} }})}}{{\left( {{{i_{0} } \mathord{\left/ {\vphantom {{i_{0} } {2g}}} \right. \kern-\nulldelimiterspace} {2g}}} \right)s^{2} - {{(i_{0} } \mathord{\left/ {\vphantom {{(i_{0} } {x_{0} )}}} \right. \kern-\nulldelimiterspace} {x_{0} )}}}}$$

According to the characteristic equation of the open-loop system, the open-loop poles of the system can be obtained as $s = \pm \sqrt {{{2g} \mathord{\left/ {\vphantom {{2g} {x_{0} }}} \right. \kern-\nulldelimiterspace} {x_{0} }}}$. It can be seen that the control system has an open loop pole located in the right half plane of the complex plane. Therefore, the magnetic levitation ball system is an open-loop unstable system.

Furthermore, let $x_{1} = U_{out}$, $x_{2} = \dot{x}_{1}$, then the state equation of the magnetic levitation ball system is as follows

10 $$\left\{ {\begin{array}{*{20}l} {\left[ {\begin{array}{*{20}c} {\dot{x}_{1} } \\ {\dot{x}_{2} } \\ \end{array} } \right] = \left[ {\begin{array}{*{20}c} 0 & 1 \\ a & 0 \\ \end{array} } \right]\left[ {\begin{array}{*{20}c} {x_{1} } \\ {x_{2} } \\ \end{array} } \right] + \left[ {\begin{array}{*{20}c} 0 \\ b \\ \end{array} } \right]U_{in} } \hfill \\ {y = \left[ {\begin{array}{*{20}c} 1 & 0 \\ \end{array} } \right]\left[ {\begin{array}{*{20}c} {x_{1} } & {x_{2} } \\ \end{array} } \right]^{ - 1} = x_{1} } \hfill \\ \end{array} } \right.$$

where $a = 2{g \mathord{\left/ {\vphantom {g {x_{0} }}} \right. \kern-\nulldelimiterspace} {x_{0} }}$, $b = - {{2gK_{s} } \mathord{\left/ {\vphantom {{2gK_{s} } {i_{0} K_{a} }}} \right. \kern-\nulldelimiterspace} {i_{0} K_{a} }}$, while $K_{s}$ and $K_{a}$ are the gains of the photoelectric position sensor and the power amplifier respectively.

Define the coefficient matrix in Eq. (10) as follows

11 $$A = \left[ {\begin{array}{*{20}c} 0 & 1 \\ a & 0 \\ \end{array} } \right],\quad B = \left[ \begin{gathered} 0 \hfill \\ b \hfill \\ \end{gathered} \right],{\kern 1pt} \quad C = \left[ \begin{gathered} 1 \hfill \\ 0 \hfill \\ \end{gathered} \right]^{T}$$

So the controllability matrix *P* and the observability matrix *Q* of the magnetic levitation ball system can be calculated by

12 $$\left\{ \begin{gathered} P = [B\, \vdots\, AB] = \left[ {\begin{array}{*{20}c} 0 & b \\ b & 0 \\ \end{array} } \right] \hfill \\ Q = \left[ \begin{gathered} C \hfill \\ CA \hfill \\ \end{gathered} \right] = \left[ {\begin{array}{*{20}c} 1 & 0 \\ 0 & 1 \\ \end{array} } \right] \hfill \\ \end{gathered} \right.$$

From Eq. (12), the rank of matrices *P* and *Q* can be obtained as follows

13 $$\left\{ \begin{gathered} Rank(P) = 2 \hfill \\ Rank(Q) = 2 \hfill \\ \end{gathered} \right.$$

As known from Eq. (13), the rank of the controllability matrix *P* is equal to the dimension of the state variables, while the rank of the observability matrix *Q* is equal to the dimension of the output vector. In other words, the magnetic levitation ball system is both controllable and observable. Consequently, the controller can be designed to make the system stable.

## Controller design

**Figure 2:**
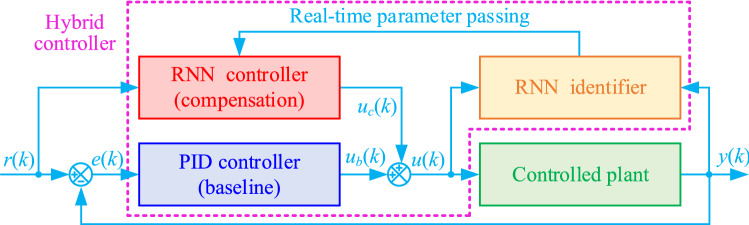
Structure diagram of the hybrid control strategy based on RNN.

In this section, a hybrid controller is designed for position control of the magnetic levitation ball system. As shown in Fig. 2, the designed controller mainly consists of a PID controller, an RNN controller, and an RNN identifier.

For the hybrid controller, the PID controller is the baseline controller, and the RNN controller is the compensation controller. Where the PID controller is mainly applied to provide online samples for the RNN identifier to learn the inverse model of the controlled object in real-time and also used to maintain the stability of the control system when the RNN is untrained at the early control phase. And the RNN controller sharing the same network structure as the RNN identifier is utilized to carry out accurate compensation control.

In addition, the RNN identifier is employed to learn the inverse model of the controlled object online, considering the historical data of the control system. And the learning parameters from the RNN identifier are passed to the RNN controller in real-time. Therefore, the proposed controller can achieve the high-precision position control of the magnetic levitation ball, benefiting from that the RNN identifier accurately learns the inverse model of the control object, and the RNN controller implements exact compensation control in the real-time.

Concretely, the control law *u*(*k*) of the hybrid controller is designed as follows

14 $$u(k) = u_{b} (k) + u_{c} (k)$$

where $u_{b} (k)$ and $u_{c} (k)$ denote the control law of PID and RNN controllers respectively.

### Baseline controller

With the characteristics of clear functionality, structure simplicity, ease of use, and applicability, the PID controller has been regarded as the simplest and yet most efficient scheme for lots of industrial control problems^34^. Consequently, this article takes the PID controller as a baseline controller in the proposed hybrid control strategy.

In practice, as a baseline controller of the hybrid control strategy, the PID controller is employed to provide the online learning samples and maintain the system stability at the early control phase, especially when the neural network is untrained. Concretely, the control law *u*_*b*_ at the moment *k* is defined as follows

15 $$u_{b} (k) = k_{p} e(k) + k_{i} \sum\limits_{i = 0}^{k} {e(i)\Delta t} + k_{d} \frac{e(k) - e(k - 1)}{{\Delta t}}$$

where $k_{p}$ is proportion gain, $k_{i}$ is integration gain, and $k_{d}$ is differentiation gain.

### RNN controller

**Figure 3:**
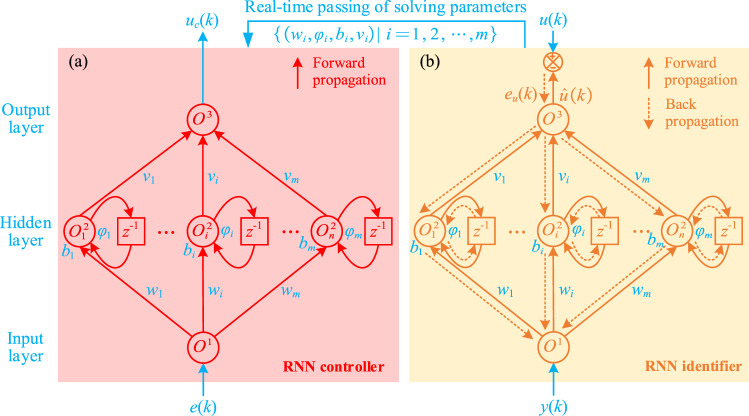
Network structures of (**a**) the RNN controller and (**b**) the RNN identifier.

As one main part of the hybrid control strategy, the RNN controller is utilized for the position compensation in the magnetic levitation ball system. Concretely, as shown in Fig. 3a, the RNN controller consists of an input layer, a hidden layer, and an output layer.

For the RNN controller, the tracking error $e(k)$ is taken as input, then the input layer at moment *k* can be defined as follows

16 $$O^{1} (k) = e(k) = r(k) - y(k)$$

The hidden layer of the RNN controller at moment *k* is calculated as follows

17 $$O_{i}^{2} (k) = \sigma (w_{i} O^{1} (k) + \varphi_{i} \sigma (k - 1) + b_{i} )$$

where $w_{i}$ denotes the *i*th weight between the input and hidden layers, $\varphi_{i}$ represents the *i*th weight between the moments *k *− 1 and *k* in the hidden layer, $b_{i}$ is the *i*th bias in the hidden layer, $\sigma ( \cdot )$ is the tanh activation function.

The output layer of the RNN controller at moment *k* is obtained as follows

18 $$O^{3} (k) = \sum\limits_{i = 1}^{n} {v_{i} O_{i}^{2} (k)}$$

where $v_{i}$ denotes the *i*th weight between the hidden and output layers.

Substitute Eqs. (16) and (17) into Eq. (18), then the control law $u_{c}$ of the RNN controller at the moment *k* can be obtained as follows

19 $$\begin{aligned} u_{c} (k) & = O^{3} (k) = \sum\limits_{i = 1}^{n} {v_{i} O_{i}^{2} (k)} \\ & {\kern 1pt} = \sum\limits_{i = 1}^{n} {v_{i} \sigma (w_{i} e(k) + \varphi_{i} \sigma (k - 1) + b_{i} )} \\ \end{aligned}$$

It is worthy to note that the RNN controller only carries out the forward propagation calculation but does not carry out the back propagation calculation, and the solving parameters ($w_{i} ,\varphi_{i} ,b_{i}$, and $v_{i}$) are real-time passed from the RNN identifier. Finally, the compensation control law can be obtained according to Eq. (19).

### RNN identifier

The RNN identifier shares the same network structure as the RNN controller. To learn the inverse model of the controlled object more accurately, the RNN identifier performs both the forward and back propagation calculations.

Similarly to the RNN controller, as shown in Fig. 3b, the forward propagation of the RNN identifier at the moment *k* can be calculated by

20 $$\begin{aligned} \hat{u}(k) & = O^{3} (k) = \sum\limits_{i = 1}^{n} {v_{i} O_{i}^{2} (k)} = \sum\limits_{i = 1}^{n} {v_{i} \sigma (z_{i} (k))} \\ & = \sum\limits_{i = 1}^{n} {v_{i} \sigma (w_{i} y(k) + \varphi_{i} \sigma (k - 1) + b_{i} )} \\ \end{aligned}$$

where $z_{i} (k) = w_{i} O^{1} (k) + \varphi_{i} \sigma (k - 1) + b_{i}$ denotes the neuron input of the hidden layer.

In the back propagation process, define ${\mathcal{L}}(k)$ the loss function at moment *k* as follows

21 $${\mathcal{L}}(k) = \frac{1}{2}(u(k) - \hat{u}(k))^{2}$$

where $u(k)$ denotes the actual control law of the hybrid controller at moment *k*, $\hat{u}(k)$ represents the estimated control law of the RNN identifier at moment *k*.

The gradient of the weight $v_{i}$ is calculated by

22 $$g_{{v_{i} }} (k) = \frac{{\partial {\mathcal{L}}(k)}}{{\partial v_{i} }} = \frac{{\partial {\mathcal{L}}(k)}}{{\partial \hat{u}(k)}}\frac{{\partial \hat{u}(k)}}{{\partial v_{i} }} = (\hat{u}(k) - u(k))O_{i}^{2} (k)$$

Let $\delta_{i} (k)$ denote the partial derivative of ${\mathcal{L}}(k)$ with respect to $z_{i} (k)$, then the formula for $\delta_{i} (k)$ is as follows

23 $$\begin{aligned} \delta_{i} (k) & = \frac{{\partial {\mathcal{L}}(k)}}{{z_{i} (k)}} = \left( {\frac{{\partial {\mathcal{L}}(k)}}{{\partial \hat{u}(k)}}\frac{{\partial \hat{u}(k)}}{{\partial O_{i}^{2} (k)}} + \frac{{\partial {\mathcal{L}}(k)}}{{\partial z_{i} (k + 1)}}\frac{{\partial z_{i} (k + 1)}}{{\partial O_{i}^{2} (k)}}} \right)\frac{{\partial O_{i}^{2} (k)}}{{\partial z_{i} (k)}} \\ & = ((\hat{u}(k) - u(k))v_{i} + \delta_{i} (k + 1)\varphi_{i} )(1 - (z_{i} (k))^{2} ) \\ \end{aligned}$$

The gradient of the weight *φ*_*i*_ is calculated by

24 $$g_{{\varphi_{i} }} (k) = \frac{{\partial {\mathcal{L}}(k)}}{{\partial \varphi_{i} }} = \frac{{\partial {\mathcal{L}}(k)}}{{\partial z_{i} (k)}}\frac{{\partial z_{i} (k)}}{{\partial \varphi_{i} }} = \delta_{i} (k)O_{i}^{2} (k - 1)$$

The gradient of the weight *b*_*i*_ is calculated by

25 $$g_{{b_{i} }} (k) = \frac{{\partial {\mathcal{L}}(k)}}{{\partial b_{i} }} = \frac{{\partial {\mathcal{L}}(k)}}{{\partial z_{i} (k)}}\frac{{\partial z_{i} (k)}}{{\partial b_{i} }} = \delta_{i} (k)$$

The gradient of the weight *w*_*i*_ is calculated by

26 $$g_{{w_{i} }} (k) = \frac{{\partial {\mathcal{L}}(k)}}{{\partial w_{i} }} = \frac{{\partial {\mathcal{L}}(k)}}{{\partial z_{i} (k)}}\frac{{\partial z_{i} (k)}}{{\partial w_{i} }} = \delta_{i} (k)O^{1} (k)$$

To simplify the parameter updating process, uniformly mark $v_{i}$, $\varphi_{i}$, $b_{i}$, and $w_{i}$ as $\theta$, then the parameters of the RNN identifier can be updated as follow

27 $$\left\{ \begin{gathered} s(k) = 0.95s(k - 1) + (g_{\theta } (k))^{2} \hfill \\ \theta (k) = \theta (k - 1) - \frac{\eta }{{\sqrt {s(k) + \varepsilon } }}\theta (k) + \gamma (g_{\theta } (k) - g_{\theta } (k - 1)) \hfill \\ \end{gathered} \right.$$

where hyperparameters $\eta$ and $\gamma$ are the learning rate and the penalty coefficient respectively, and the smoothing term $\varepsilon = 1e - 8$ is used to avoid division by zero.

## Simulation analysis

### Simulation setup

To demonstrate the effectiveness of the designed controller, the simulation tests are carried out on the Matlab/Simulink. For the magnetic levitation ball system investigated in this article, the physical parameters are described in Table 1.

**Table 1:** The physical parameters of the magnetic levitation ball system.

Physical descriptions	Parameters (unit)	Nominal values
Mass of the steel ball	m (kg)	0.022
Equilibrium point	*x*_0_/mm	20
Current at equilibrium point	*i*_0_/mm	0.6105
Gain of the position sensor	*K*_s_	− 458.7156
Gain of the power amplifier	*K*_a_	5.8929

Substitute the parameters described in Table 1 into Eq. (5), the numerical transfer function of the controlled object can be described as follows

28 $$G(s) = \frac{77.8421}{{0.0311s^{2} { - }30.5250}}$$

Based on the numerical transfer function (28), the baseline controller based on PID (PID), the hybrid controller based on PID and BPNN (PID + BPNN), and the hybrid controller based on PID and RNN (PID + RNN) are quantitatively compared on the following measurements, namely, the mean absolute error (MAE), the root-mean-square error (RMSE), the integral time absolute error (ITAE), and the integral time square error (ITSE). The formulations of these four measurements are expressed as follows

29 $$\left\{ \begin{gathered} MAE = \frac{1}{T}\sum\limits_{t = 0}^{T} {\left| {r(t) - y(t)} \right|} ,\quad RMSE = \sqrt {\frac{1}{T}\sum\limits_{t = 0}^{T} {(r(t) - y(t))^{2} } } \hfill \\ ITAE = \int_{0}^{T} {t\left| {r(t) - y(t)} \right|dt,} \quad ITSE = \int_{0}^{T} {t(r(t) - y(t))^{2} dt} \hfill \\ \end{gathered} \right.$$

**Figure 4:**
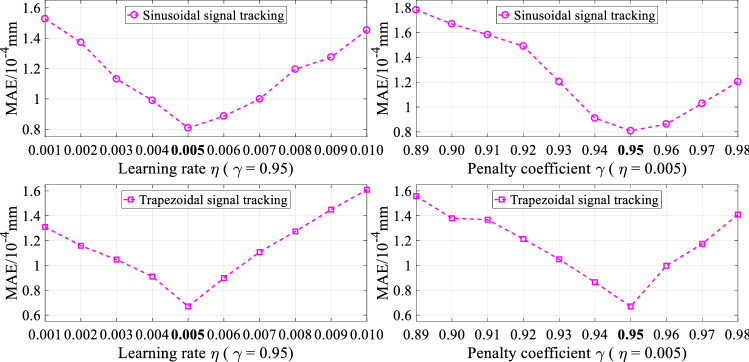
Simulation results of the RNN-based hybrid controller under different hyper-parameters.

The tracking controls of the step, square, sinusoidal, and trapezoidal signals are implemented in the simulation tests. In particular, for the tracking controls of step and square signals, the overshoot and settling time are considered besides MAE and RMSE. For the tracking controls sinusoidal and trapezoidal signals, the steady state error is considered besides MAE and RMSE. Moreover, for the fair comparison of the different control strategies, we try our best to make control performance to be the best by adjusting the appropriate parameters of each controller. Take the tracking controls of sinusoidal and trapezoidal signals as examples, the simulation results of the RNN-based hybrid controller under different hyper-parameters are shown in Fig. 4.

Ultimately, for the simulation tests, the optimal parameters of the different controllers are described in Table 2.

**Table 2:** The optimal parameters of the different controllers in the simulation tests.

Controller	All signals				Step		Square		Sinusoidal		Trapezoidal	
	*K*_*p*_	*K*_*i*_	*K*_*d*_	*m*	*η*	*γ*	*η*	*γ*	*η*	*γ*	*η*	*γ*
PID	8	2	0.6	–	–	–	–	–	–	–	–	–
PID+BPNN	8	2	0.6	5	0.010	0.95	0.045	0.90	0.007	0.90	0.007	0.90
PID+RNN	8	2	0.6	5	0.008	0.95	0.015	0.95	0.005	0.95	0.005	0.95

### Tracking performance

**Figure 5:**
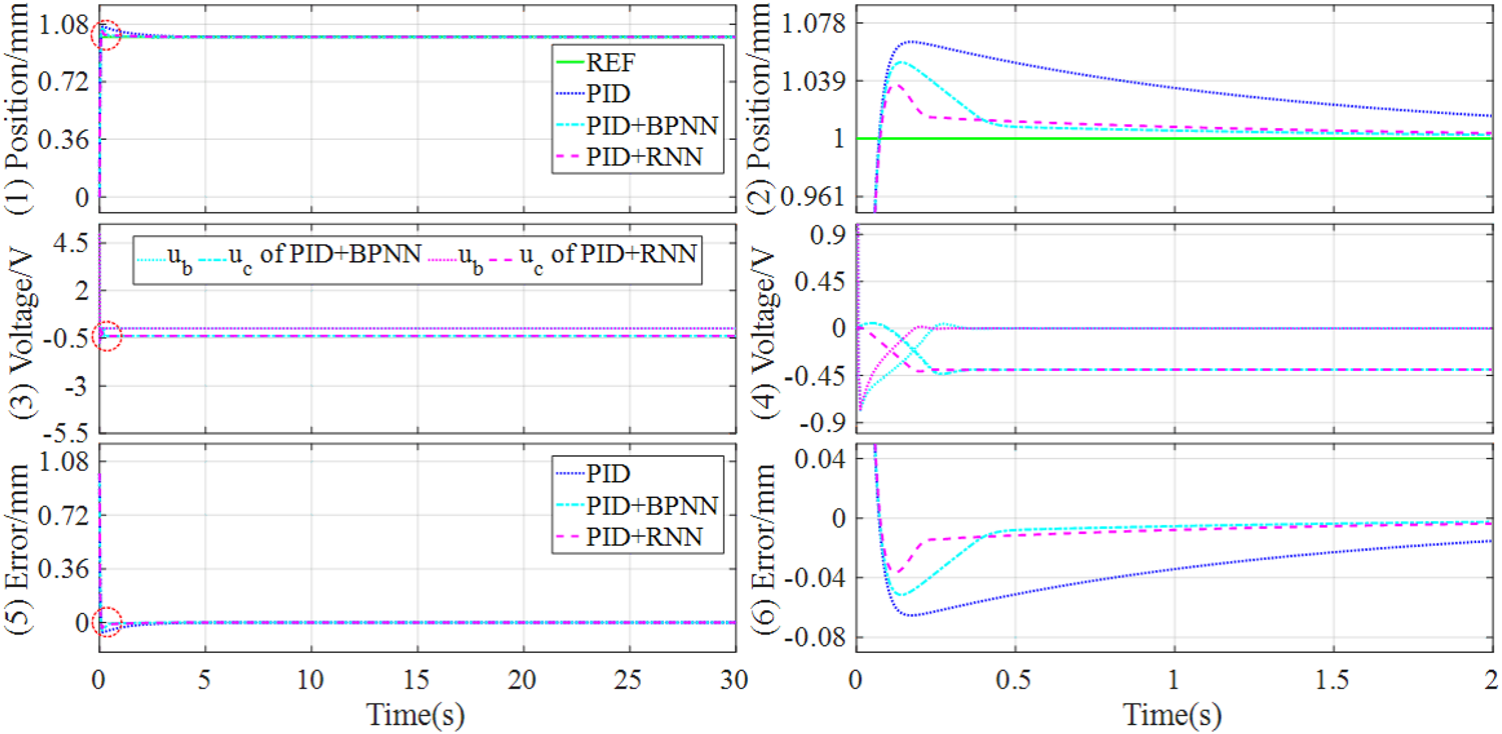
Simulation results of tracking step signal under the different controllers.

**Figure 6:**
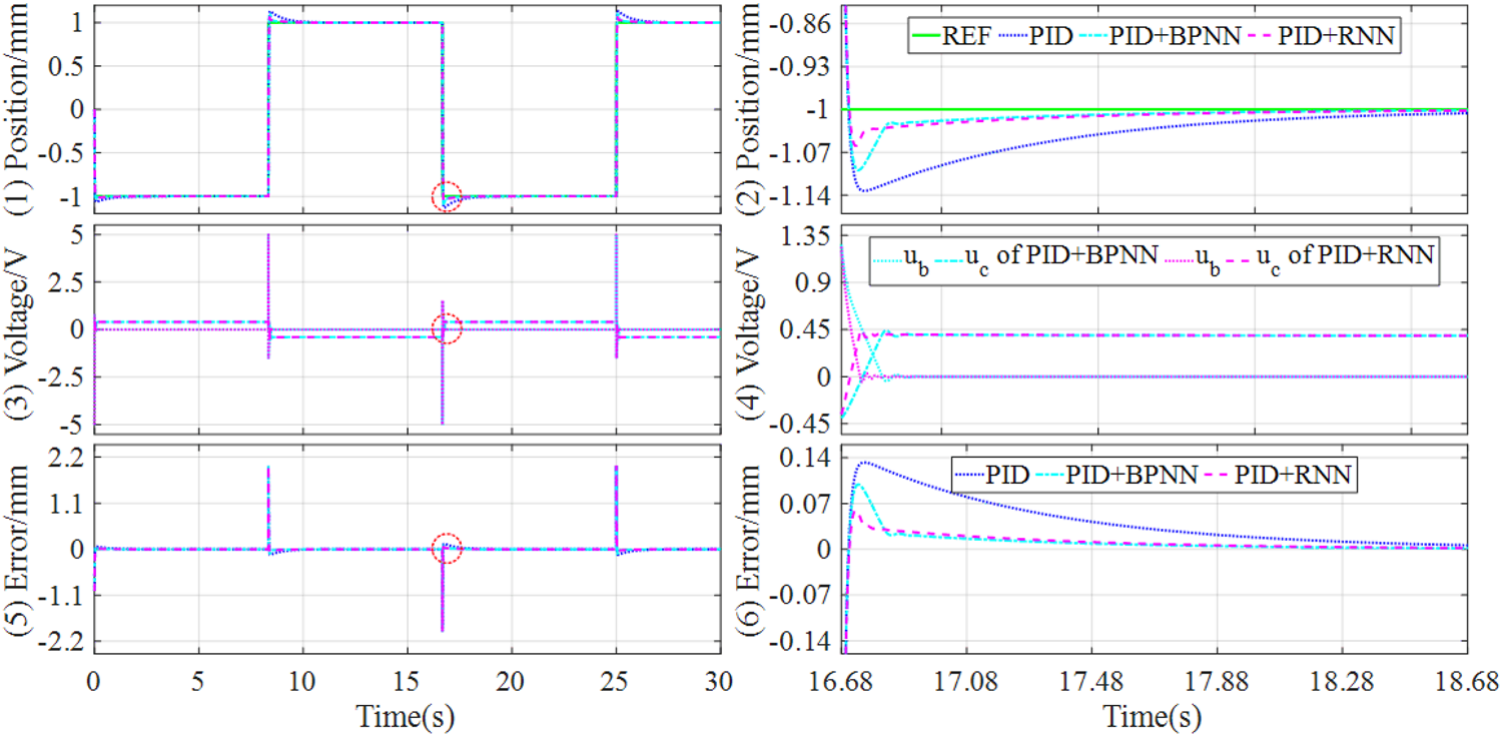
Simulation results of tracking square signal under the different controllers.

For the simulation tests, the control responses of tracking step and square signals under the different comparison controllers are presented in Figs. 5 and 6, where the subgraph on the left is the enlarged version in the red circle of the corresponding subgraph on the right. The performance evaluations of these tracking controls are described in Table 3.

**Table 3:** Comparisons of simulation results of tracking step and square signal.

Signal	Controller	Overshoot	Settling time	MAE	RMSE	ITAE	ITSE
Step (Fig. 5)	PID	0.0652	0.8325	0.0037	0.0243	0.1178	0.0024
	PID + BPNN	0.0556	0.2704	0.0016	0.0225	0.0296	2.7443 × 10^–4^
	PID + RNN	0.0364	0.1915	0.0015	0.0223	0.0244	2.6702 × 10^–4^
	Improvement	34.53%	29.18%	6.25%	0.89%	17.57%	2.70%
Square (Fig. 6)	PID	0.1327	0.7050	0.0132	0.0627	0.5892	0.1824
	PID + BPNN	0.1022	0.1299	0.0056	0.0573	0.2467	0.1518
	PID + RNN	0.0625	0.0754	0.0055	0.0570	0.2440	0.1504
	Improvement	35.85%	41.96%	1.79%	0.52%	1.09%	0.92%

As shown in Figs. 5 and 6, three controllers all can realize the tracking control of the magnetic levitation ball, but the separate PID controller has a larger overshoot and a longer settling time. On the contrary, the hybrid controllers can lower the overshoot and shorten the settling time of the control responses to some extent. Moreover, the hybrid controller based on PID and RNN can obtain the best dynamic quality by further lowing the overshoot and shortening the settling time of the control responses, compared with the hybrid controller based on PID and BPNN.

Concretely, as shown in Table 5, for step signal tracking control, the RNN-based hybrid controller reduces overshoot by 34.53% and the settling time by 29.18%, compared with the BPNN-based hybrid controller. For square signal tracking control, the RNN-based hybrid controller reduces the overshoot and settling time by 35.85% and 41.96% respectively, compared with the BPNN-based hybrid controller. Therefore, the RNN-based hybrid controller can obtain excellent dynamic performances in the simulation of tracking step and square signals.

**Figure 7:**
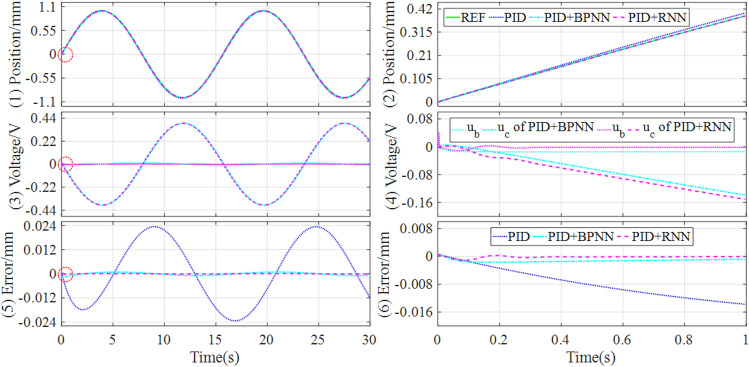
Simulation results of tracking sinusoidal signal under the different controllers.

**Figure 8:**
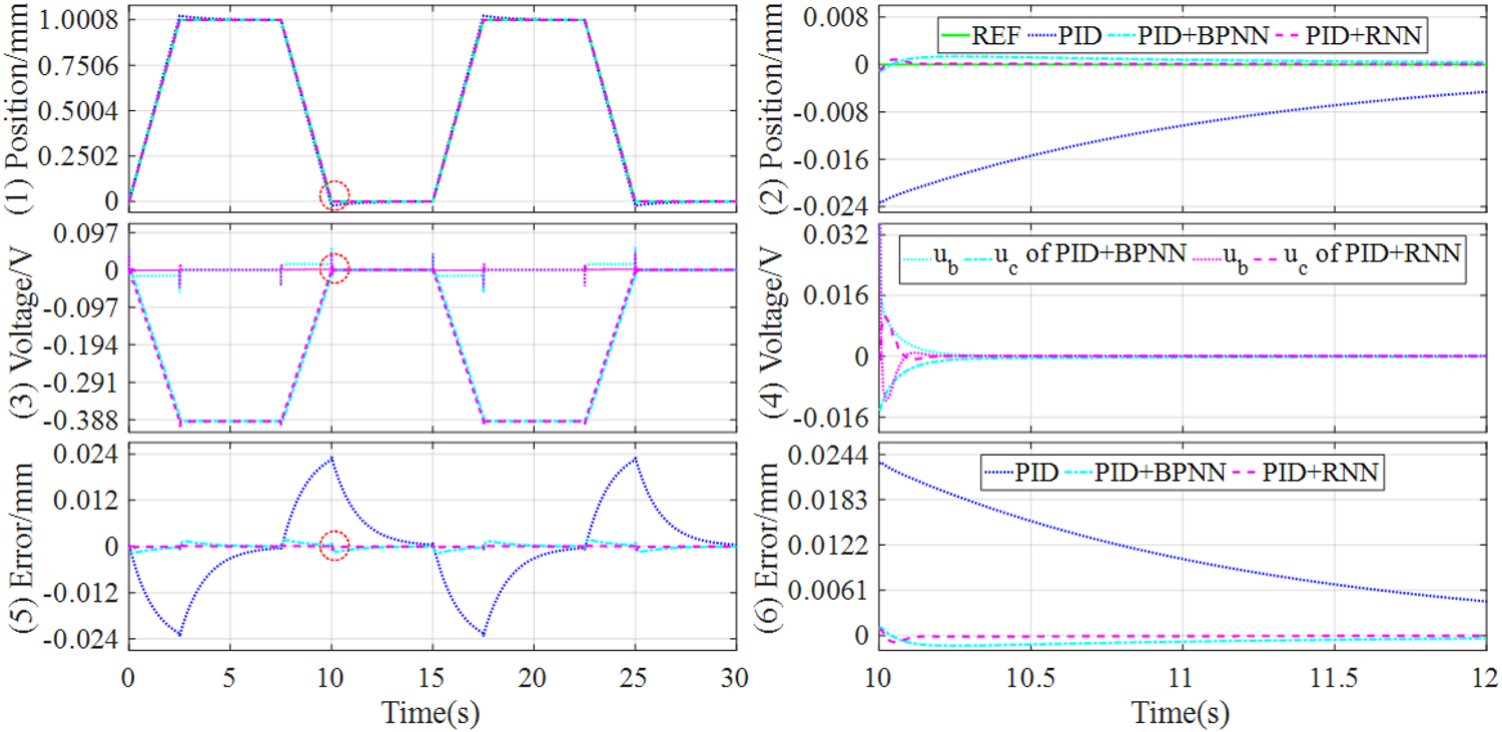
Simulation results of tracking trapezoidal signal under the different controllers.

To further validate the control performances, the tracking controls of sinusoidal and trapezoidal signals are simulated, the control responses are drawn in Figs. 7 and 8, and the performance evaluations of the tracking controls are described in Table 4.

**Table 4:** Simulation comparisons of tracking sinusoidal and trapezoidal signals.

Signal	Controller	Steady state error	MAE	RMSE	ITAE	ITSE
Sinusoidal (Fig. 7)	PID	[− 2.3433, 2.3418] × 10^–2^	1.3961 × 10^–2^	1.5677 × 10^–2^	3.1567	0.0565
	PID + BPNN	[− 8.6829, 8.6830] × 10^–4^	5.9044 × 10^–4^	6.5988 × 10^–4^	0.1321	9.3511 × 10^–5^
	PID + RNN	[− 1.1400, 1.1158] × 10^–4^	8.0844 × 10^–5^	1.0025 × 10^–4^	0.0179	1.6445 × 10^–6^
	Improvement	86.87%, 87.15%	86.31%	84.81%	86.45%	98.24%
Trapezoidal (Fig. 8)	PID	[− 2.3411, 2.3356] × 10^–1^	8.5593 × 10^–3^	1.1346 × 10^–2^	3.5357	0.0518
	PID + BPNN	[− 1.6541, 1.5965] × 10^–3^	5.6145 × 10^–4^	7.1995 × 10^–4^	0.2293	2.0268 × 10^–4^
	PID + RNN	[− 8.7459, 8.5966] × 10^–4^	6.6979 × 10^–5^	1.1852 × 10^–4^	0.0271	4.9290 × 10^–6^
	Improvement	47.13%, 46.15%	88.07%	83.54%	88.18%	97.57%

It can be known from Figs. 7 and 8 that, the hybrid controllers can improve the control response and lower the tracking error, compared with the separate baseline controller based on PID. In addition, the hybrid controller based on PID and RNN obtains the best control quality and the lowest tracking error in all comparison controllers, benefiting from the RNN identifier builds a more accurate inverse model of the controlled object.

From Table 4, it can be found that when tracking sinusoidal signal, the hybrid controller based on PID and RNN lowers the control error above 80%, compared with the hybrid controller based on PID and BPNN. When tracking trapezoidal signal, the hybrid controller based on PID and RNN lowers the steady-state error, MAE, and RMSE above 40%, 80%, and 80% respectively, compared with the hybrid controller based on PID and BPNN. Consequently, whether tracking sinusoidal signal or trapezoidal signal, the RNN-based hybrid controller can significantly improve the control performances compared with the BPNN-based hybrid controller.

## Experimental verification

### Experimental setup

**Figure 9:**
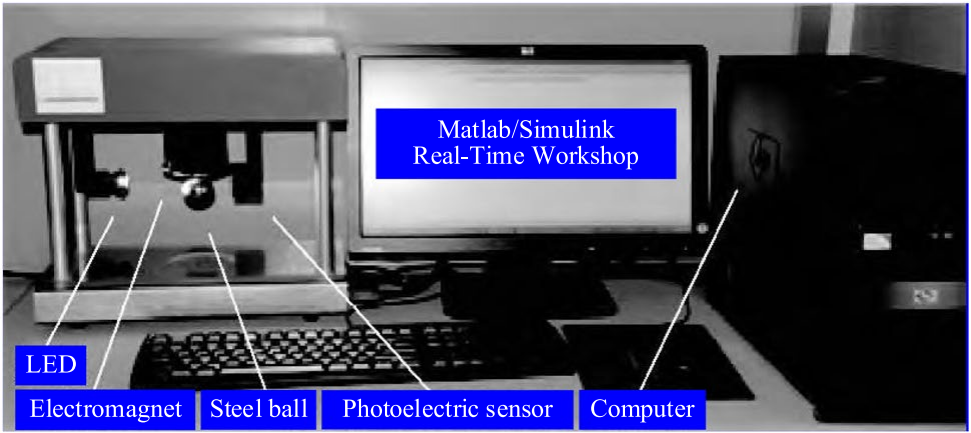
Experimental verification platform of the magnetic levitation ball system.

To further verify the effectiveness and the advancement of the RNN-based hybrid controller, the hardware-in-loop platform shown in Fig. 9 is used to conduct experimental research on the position control of the magnetic levitation ball system. The tracking controls of the step, square, sinusoidal, and trapezoidal signals are carried out in the Matlab/Simulink real-time workshop.

**Figure 10:**
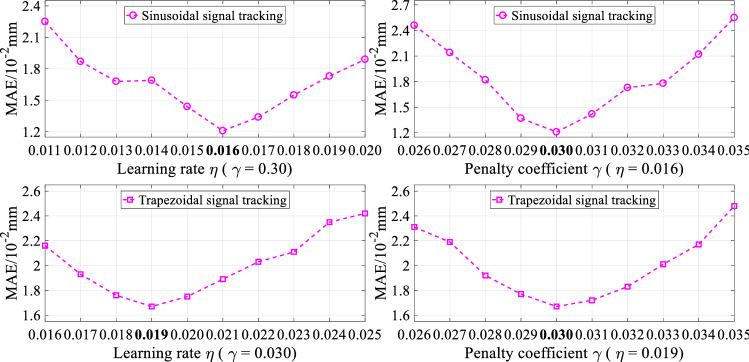
Experiment results of the RNN-based hybrid controller under different hyper-parameters.

In the comparison controllers, hyper-parameters play a critical role in controller performance. Take the tracking controls of sinusoidal and trapezoidal signals as examples, the experiment results of the RNN-based hybrid controller under different hyper-parameters are displayed in Fig. 10. Therefore, for the fair comparison of the different control schemes in the experimental tests, we also try our best to make control performance to be the best by adjusting the appropriate parameters of each controller. Specifically, for the experiment tests, the optimal parameters of the different controllers are described in Table 5.

**Table 5:** The optimal parameters of the different controllers in the experiment tests.

Controller	All signals				Step		Square		Trapezoidal		Sinusoidal	
	*K*_*p*_	*K*_*i*_	*K*_*d*_	*m*	*η*	*γ*	*η*	*γ*	*η*	*γ*	*η*	*γ*
PID	1.005	0.003	12	–	–	–	–	–	–	–	–	–
PID + BPNN	1.005	0.003	12	5	0.035	0.038	0.035	0.032	0.020	0.030	0.020	0.030
PID + RNN	1.005	0.003	12	5	0.030	0.040	0.030	0.040	0.019	0.030	0.016	0.030

### Tracking performance

**Figure 11:**
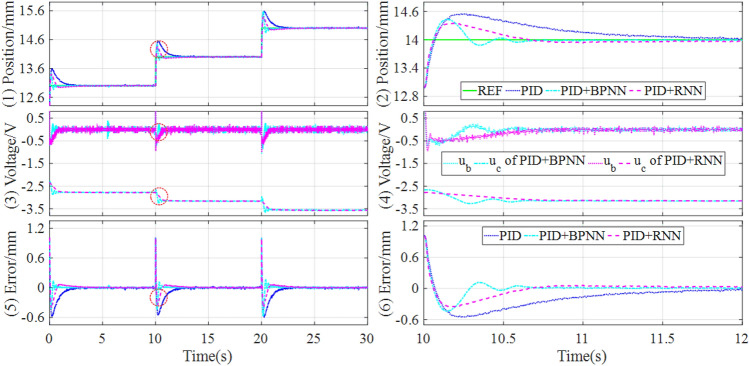
Experimental results of tracking continuous step signal under the different controllers.

**Figure 12:**
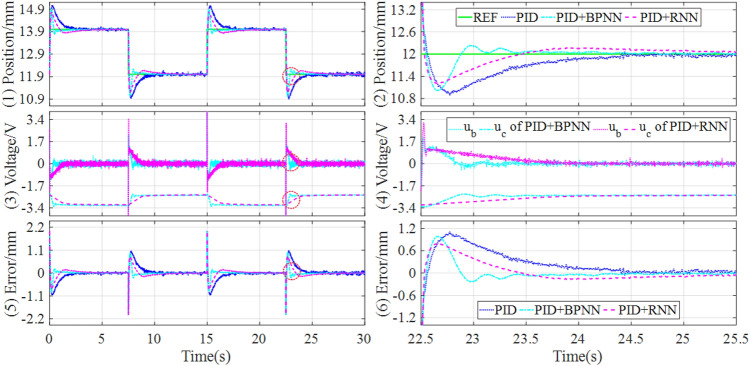
Experimental results of tracking square signal under the different controllers.

Firstly, in the experiment tests, the control response comparisons of tracking continuous step and square signals under the different controllers are shown in Figs. 11 and 12.

It can be learned from Figs. 9 and 10 that the hybrid controller can achieve lower overshoot and shorten settling time compared with the baseline controller when tracking continuous step and square signals. And the hybrid controller based on PID and RNN can acquire the best control quality among all the comparative controllers. Concretely, the performance evaluations of these tracking controls are quantitatively described in Table 6.

**Table 6:** Comparisons of experiment results of tracking step and square signal.

Signal	Controller	Overshoot	Settling time	Steady state error	MAE	RMSE	ITAE	ITSE
Step (Fig. 11)	PID	0.5576	1.6295	[− 0.0410, 0.0371]	0.0514	0.1306	5.7839	1.7809
	PID + BPNN	0.4355	0.5912	[− 0.0264, 0.0273]	0.0198	0.0768	2.9815	0.6223
	PID + RNN	0.3526	0.5645	[− 0.0166, 0.0176]	0.0185	0.0731	2.5911	0.5892
	Improvement	19.03%	4.52%	37.12%, 35.53%	6.57%	5.06%	13.09%	5.32%
Square (Fig. 12)	PID	1.0755	4.3692	[− 0.1455, 0.1230]	0.1360	0.2892	17.0246	9.6947
	PID + BPNN	0.9725	2.9880	[− 0.0381, 0.0352]	0.0571	0.2025	11.8288	4.8578
	PID + RNN	0.7821	2.8792	[− 0.0332, 0.0156]	0.0540	0.1918	9.0329	3.8641
	Improvement	19.58%	3.64%	12.86%, 55.68%	5.43%	5.28%	23.64%	20.46%

As shown in Table 6, for both continuous step and square signals, the RNN-based hybrid controller has an obvious reduction in terms of overshoot and settling time compared with the BPNN-based hybrid controller. Especially, in terms of the steady-state error, the RNN-based hybrid controller has an improvement of 37.12% and 35.53% for continuous step signal, and 12.86% and 55.68% for square signal, compared with the BPNN-based hybrid controller. As a result, through the comparative analysis of the experimental results, it can be easily found that the RNN-based hybrid controller can improve both dynamic and steady state performances.

**Figure 13:**
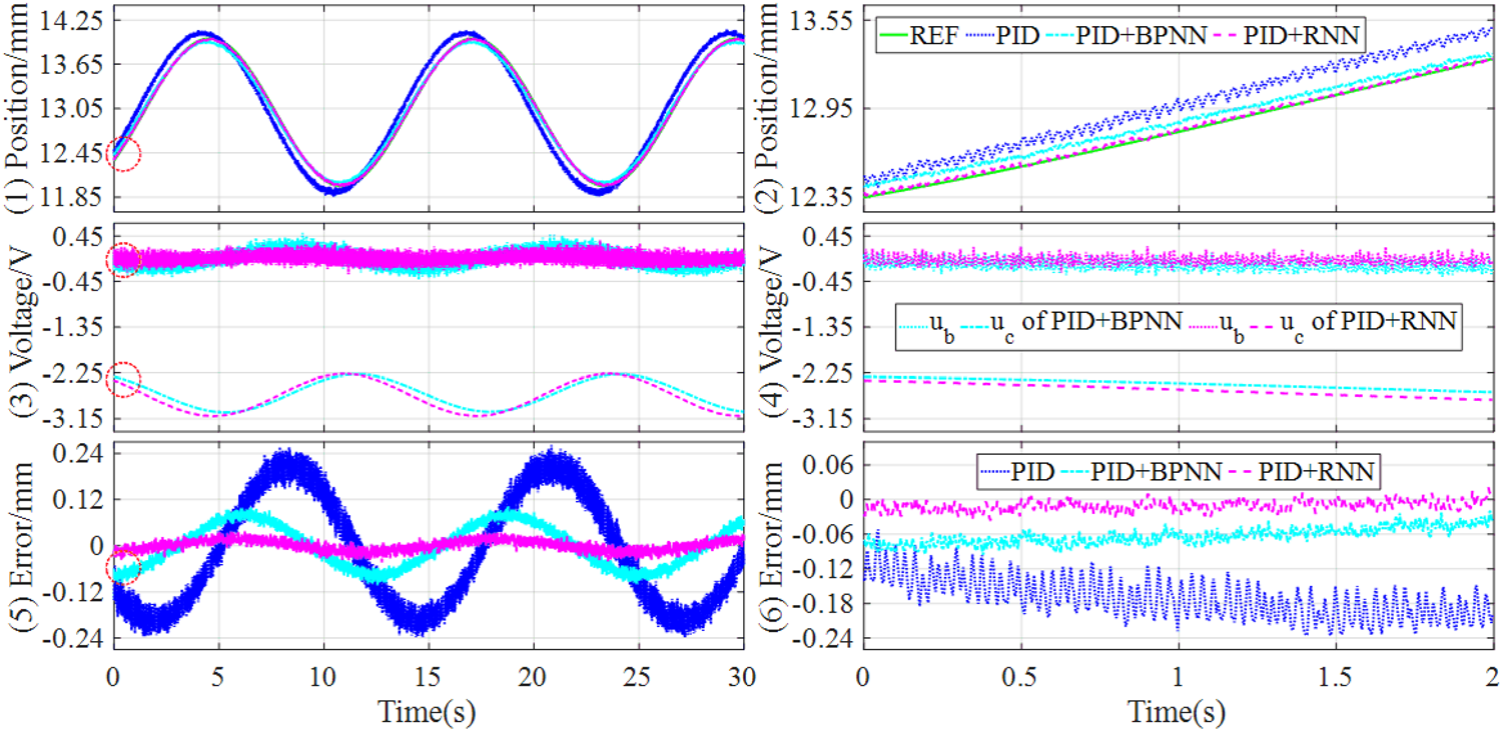
Experimental results of tracking sinusoidal signal under the different controllers.

**Figure 14:**
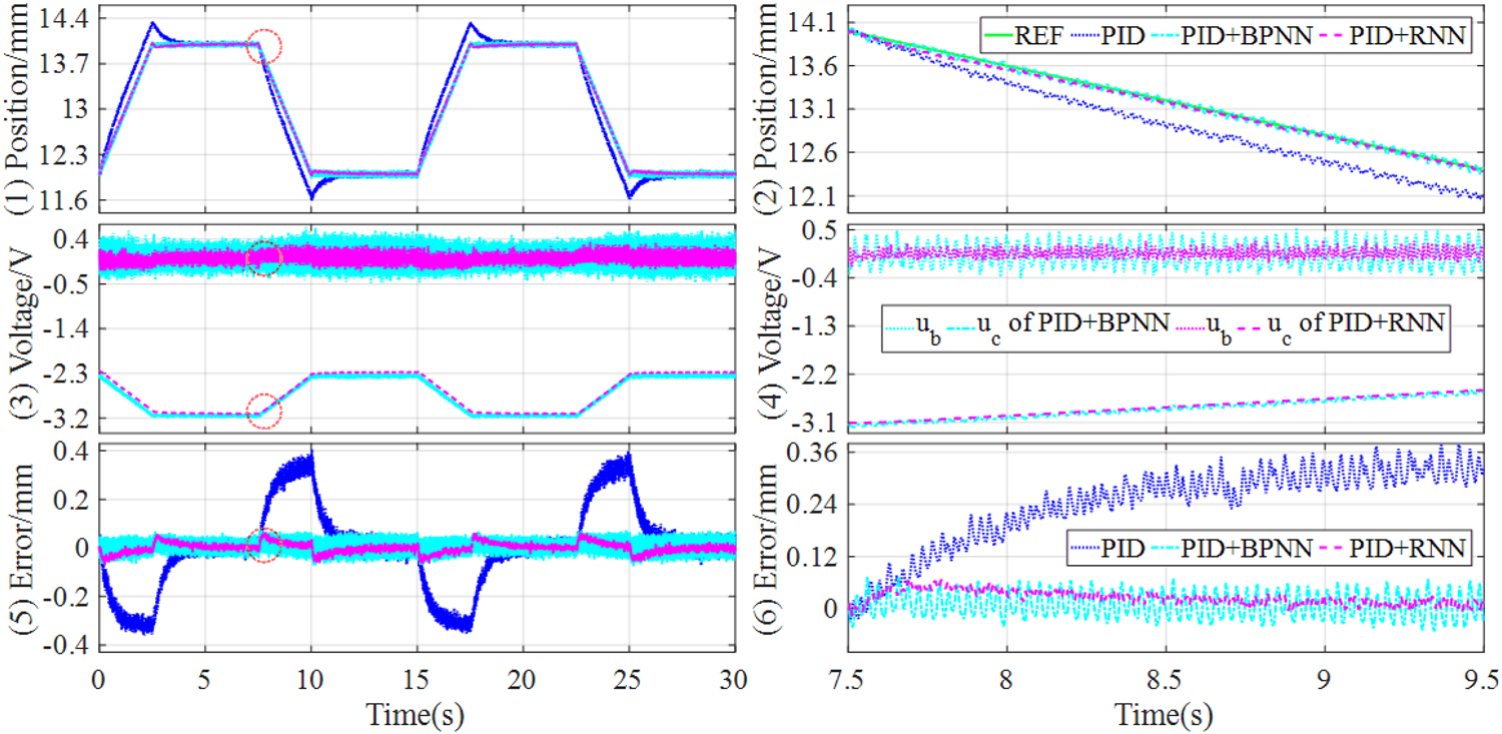
Experimental results of tracking trapezoidal signal under the different controllers.

In addition, to preferably demonstrate the control performances, the tracking controls of sinusoidal and trapezoidal signals are tested in the experiment platform, and the control responses are drawn in Figs. 13 and 14.

It can be seen that the hybrid controllers can track the reference signals better and have lower steady-state error compared with the baseline controller. Particularly, the position tracking accuracy of the magnetic levitation ball is higher than the BPNN-based hybrid controller when applying the RNN-based hybrid controller. Concretely, the performance evaluations of the tracking controls are quantitatively described in Table 7.

**Table 7:** Experiment comparisons of tracking sinusoidal and trapezoidal signals.

Signal	Controller	Steady state error	MAE	RMSE	ITAE	ITSE
Sinusoidal (Fig. 13)	PID	[− 0.2385, 0.2611]	0.1324	0.1462	19.5899	3.1416
	PID + BPNN	[− 0.1003, 0.1030]	0.0484	0.0545	7.1118	0.4307
	PID + RNN	[− 0.0412, 0.0376]	0.0121	0.0145	1.8041	0.0314
	Improvement	58.92%, 63.50%	75.00%	73.40%	74.63%	92.71%
Trapezoidal (Fig. 14)	PID	[− 0.3533, 0.3991]	0.1182	0.1706	15.823	3.7861
	PID + BPNN	[− 0.0723, 0.0742]	0.0273	0.0316	4.1962	0.1560
	PID + RNN	[− 0.0576, 0.0480]	0.0167	0.0217	2.3349	0.0629
	Improvement	20.33%, 35.31%	38.83%	31.33%	44.36%	59.68%

From Table 7, it can be found that for tracking sinusoidal and trapezoidal signals, the RNN-based hybrid controller lowers the steady state error by about 60% and 20% respectively, compared with the BPNN-based hybrid controller. Meanwhile, the RNN-based hybrid controller reduces the MAE and RMSE by above 70% and 30% respectively, compared with the BPNN-based hybrid controller. Consequently, the RNN-based hybrid controller has not only the best dynamic tracking performance but also the lowest steady state error among all the comparison controllers.

### Robust performance

**Figure 15:**
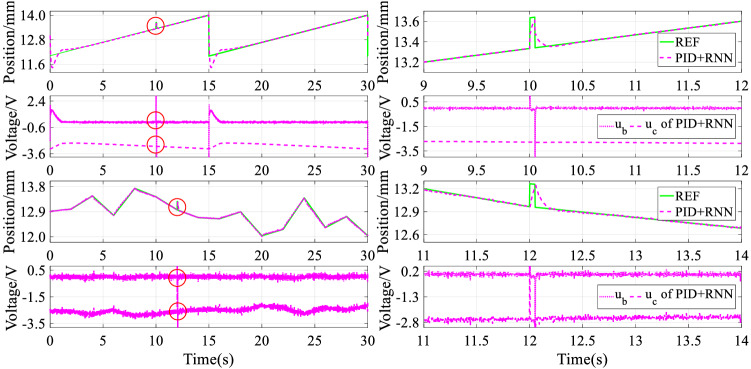
Experiment results of disturbance suppression under sawtooth and stochastic signals.

In this section, the tracking control experiments of both sawtooth and stochastic signals with constant disturbance are first utilized to indicate the disturbance rejection ability of the RNN-based intelligent controller. The experiment results are drawn in Fig. 15, where the four subgraphs above show the anti-interference performance under tracking sawtooth signal, and the four subgraphs below display the anti-interference performance under tracking stochastic signal.

It can be found from Fig. 15 that whether tracking a sawtooth or stochastic signal, the magnetic levitation ball can respond to the disturbing reference quickly. Thus the RNN-based intelligent controller can suppress disturbance effectively because of the quick response and accurate compensation from the RNN-based inverse model controller in real-time.

**Figure 16:**
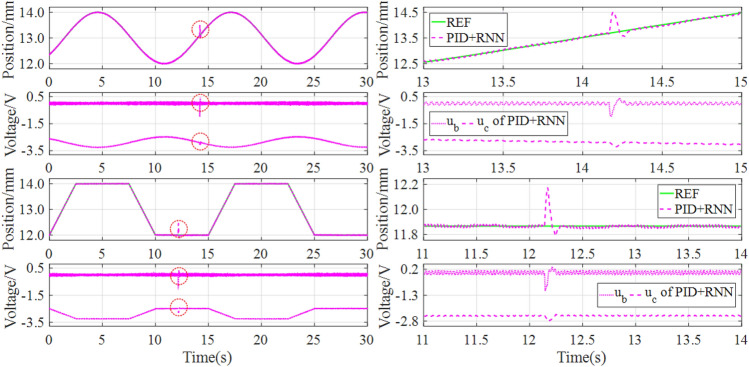
Experiment results of disturbance suppression under sinusoidal and trapezoidal signals.

In addition, the tracking control experiments of both sinusoidal and trapezoidal signals with stochastic disturbance are further employed to demonstrate the disturbance rejection ability of the RNN-based hybrid controller. The experiment results are drawn in Fig. 16, where the four subgraphs above denote the anti-interference performance under tracking sinusoidal signal, and the four subgraphs below represent the anti-interference performance under tracking sinusoidal signal.

It can be seen from Fig. 16 that whether tracking sinusoidal or trapezoidal signal, the disturbed magnetic levitation ball can track the desired reference quickly, benefiting from the RNN-based hybrid controller that can suppress disturbance effectively. Consequently, the closed-loop control system has strong robustness to some extent when using the RNN-based hybrid controller.

## Discussion

The magnetic levitation ball system is a typical nonlinear and unstable system. For improving its control accuracy and tracking performance, this study presents an intelligent controller based on a PID-based baseline controller, an RNN identifier, and an RNN controller. The RNN identifier is designed to learn the inverse dynamics model of the controlled object online then the learning parameters are passed to the RNN controller in real-time, carrying out an accurate compensation control. And the comprehensive simulation and experiment results show that the proposed controller can improve the transient performance and lower the steady-state error with excellent robustness compared with the traditional PID controller and BPNN-based internal model controller.

To further illustrate the effectiveness and the advancement of the designed controller, some advanced control approaches of the magnetic levitation ball system are experimentally compared in this section. It is worth noticing that all comparisons are performed on the same or similar experimental platform. In addition, the different methods are not compared under the time integral evaluation metric, considering the different duration of the tracking experiment.

First, as one of the effectively intelligent control approaches^35^, fuzzy control is considered to compare with the proposed controller. The experiment results of the tracking step signal show that the fluctuation range of tracking error is [− 0.05, 0.05] using the Takagi–Sugeno fuzzy controller (TSFC)^36^. The error range of the TSFC is obviously larger than that of the proposed controller, which can obtain the error range of [− 0.0166, 0.0176] when tracking the step signal.

Then, an advanced sliding mode control method named adaptive nonsingular terminal sliding mode control (ANTSMC) based on reduced order generalized proportional integral observer (RGPIO)^7^ is employed to compare with the proposed controller. As shown in Table 8, the RMSEs of the ANTSMC + RGPIO are 0.2974 and 0.1367 respectively when tracking square and sinusoidal signals, which are larger than those of the proposed PID + RNN controller.

**Table 8:** Experiment comparisons of tracking signals under the different controllers.

Signals	Controller	Steady state error	MAE	RMSE
Step	TSFC^36^	[− 0.05, 0.05]	–	–
	FI-NNCC^26^	[− 0.02, 0.02]	–	–
	PID + RNN	[− 0.0166, 0.0176]	0.0185	0.0731
Square	ANTSMC + RGPIO^7^	-	–	0.2974
	FI-NNCC^26^	[− 0.03, 0.03]	–	–
	PID + RNN	[− 0.0332, 0.0156]	0.0540	0.1918
Sinusoidal	ANTSMC + RGPIO^7^	–	–	0.1367
	NARX-NNC^22^	–	–	0.0472
	PID + RNN	[− 0.0412, 0.0376]	0.0121	0.0145

Finally, a nonlinear autoregressive exogenous-type neural network controller (NARX-NNC)^22^ is utilized to compare with the proposed controller. For the sinusoidal signal tracking, the RMSE using NNC is 0.0472, which is obviously larger than the 0.0145 obtained by the proposed controller. Besides, the fuzzy inference-based neural network compensation controller (FI-NNCC)^26^ is applied to compare with the proposed controller. As shown in Table 8, the steady state error of the FI-NNCC is a little bit larger compared with the proposed controller overall.

As a result, it can be seen from the above comparative analysis that the proposed intelligent controller based on RNN has better control performance and tracking accuracy than other advanced control approaches to the magnetic levitation ball system.

## Conclusion

In this study, the hybrid control based on RNN is proposed to achieve the high-precision position control of the magnetic levitation ball system. The main contribution of this study is that the control quantity can be adaptively compensated with high precision by the hybrid controller without establishing an accurate mathematical model of the control system. Moreover, the innovation is that the RNN controller implements high-precision control while the RNN identifier accurately learns the inverse model of the controlled object online, considering the historical data of the control system. Ultimately, the simulation and experiment results demonstrate that the RNN-based hybrid controller can improve the transient performance and lower the steady-state error with certain robustness compared with the BPNN-based hybrid controller.

The proposed RNN-based hybrid scheme has been successfully applied to the magnetic levitation ball system with a single-degree-of-freedom. And the research in this article may provide some helpful references for the application of the proposed approach in the real-time control process. In addition, the proposed hybrid controller can facilitate deployment to the second-order control system with nonlinear, time-varying, and uncertain characteristics. In future work, on the one hand, we will try to design the hybrid controller based on RNN to the multi-degree of the freedom control system; on the other hand, we will try to study the high order control problem by using an RNN-based hybrid control strategy.

## Data Availability

All data generated or analyzed during this study are included in this published article.
